# Supplementary material for: Splice-Junction-Based Mapping of Alternative Isoforms in the Human Proteome
Source: Cell Rep. Author manuscript; Available in PMC 2020 Jan 15. (PMC6961840; doi:10.1016/j.celrep.2019.11.026)
Supplement: 2 [file NIHMS1546469-supplement-2.zip › DF1/PXD009737/TestisTrypsin/TestisTrypsin_13_UGGT1_KDDSFIRPVTFWIVGDFDSPSGR.pdf]

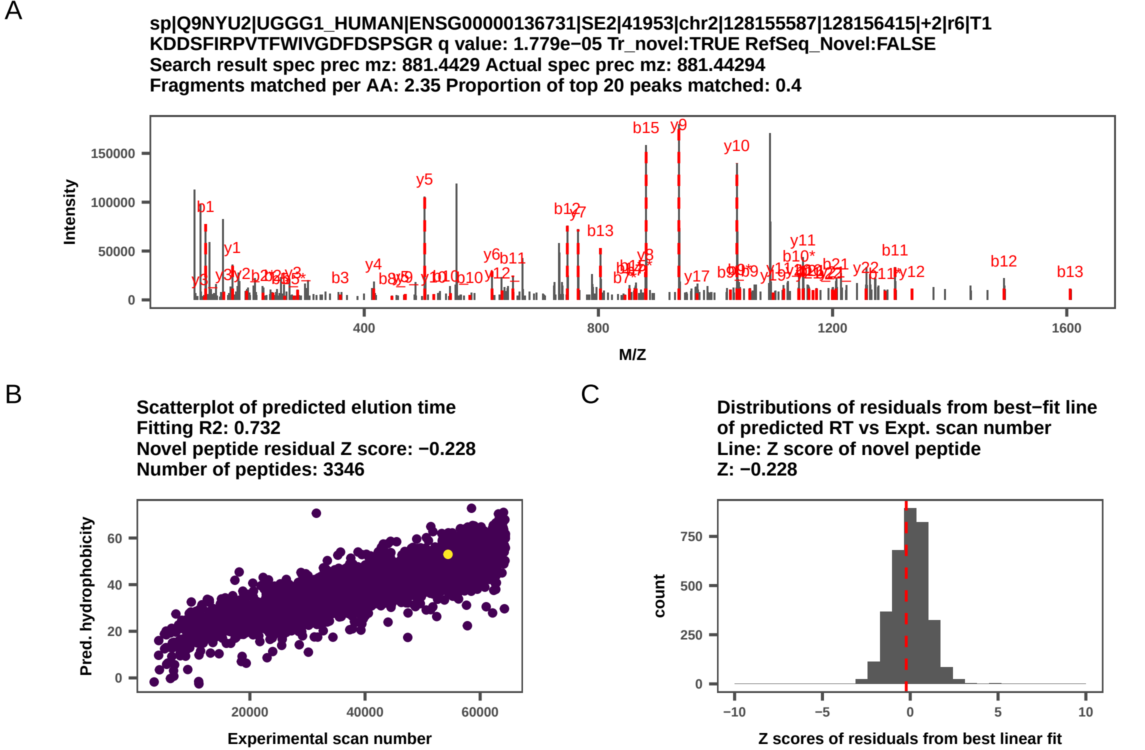
A
sp|Q9NYU2|UGGG1_HUMAN|ENSG00000136731|SE2|41953|chr2|128155587|128156415|+2|r6|T1
KDDSFIRPVTFWIVGDFDSPSGR q value: 1.779e−05 Tr_novel:TRUE RefSeq_Novel:FALSE
Search result spec prec mz: 881.4429 Actual spec prec mz: 881.44294
Fragments matched per AA: 2.35 Proportion of top 20 peaks matched: 0.4
Intensity
150000
100000
50000
0
400
800
1200
1600
M/Z
b15
y9
y10
y5
b1
y1
b13
y4
b3
y6
b11
y8
y17
y11
b10*
b21
y22
b11
y12
b12
b13
B
Scatterplot of predicted elution time
Fitting R2: 0.732
Novel peptide residual Z score: −0.228
Number of peptides: 3346
Pred. hydrophobicity
60
40
20
0
20000
40000
60000
Experimental scan number
C
Distributions of residuals from best−fit line
of predicted RT vs Expt. scan number
Line: Z score of novel peptide
Z: −0.228
count
750
500
250
0
−10
−5
0
5
10
Z scores of residuals from best linear fit
